# A Deep Learning-Based Model for Classifying Osteoporotic Lumbar Vertebral Fractures on Radiographs: A Retrospective Model Development and Validation Study

**DOI:** 10.3390/jimaging9090187

**Published:** 2023-09-18

**Authors:** Yohei Ono, Nobuaki Suzuki, Ryosuke Sakano, Yasuka Kikuchi, Tasuku Kimura, Kenneth Sutherland, Tamotsu Kamishima

**Affiliations:** 1 Department of Radiology, NTT East Medical Center Sapporo, South-1 West-15, Chuo-Ku, Sapporo 060-0061, Japan; yohei.ono32@gmail.com (Y.O.); n-suzuki@east.ntt.co.jp (N.S.); 2 Graduate School of Health Sciences, Hokkaido University, North-12 West-5, Kita-Ku, Sapporo 060-0812, Japan; 3 Department of Radiological Technology, Hokkaido University Hospital, Kita-14 Nishi-5, Kita-Ku, Sapporo 060-8648, Japan; r.sakano@huhp.hokudai.ac.jp; 4 Department of Diagnostic Imaging, Faculty of Medicine, Hokkaido University, Kita-15 Nishi-7, Kita-Ku, Sapporo 060-8638, Japan; 5 Department of Diagnostic and Interventional Radiology, Tonan Hospital, Kita 4 Nishi 7, Chuo-Ku, Sapporo 060-0004, Japan; yasuka.kikuchi@tonan.gr.jp; 6 Department of Radiology, Hokkaido Medical Center, Yamanote5-7, Nishi-Ku, Sapporo 063-0005, Japan; task1006@gmail.com; 7 Global Center for Biomedical Science and Engineering, Hokkaido University, North-15 West-7, Kita-Ku, Sapporo 060-8638, Japan; kenneth.lee.sutherland@gmail.com; 8 Faculty of Health Sciences, Hokkaido University, North-12 West-5, Kita-Ku, Sapporo 060-0812, Japan

**Keywords:** osteoporotic vertebral fractures, radiography, deep learning, convolutional neural networks, computer-aided diagnosis, automatic classification

## Abstract

Early diagnosis and initiation of treatment for fresh osteoporotic lumbar vertebral fractures (OLVF) are crucial. Magnetic resonance imaging (MRI) is generally performed to differentiate between fresh and old OLVF. However, MRIs can be intolerable for patients with severe back pain. Furthermore, it is difficult to perform in an emergency. MRI should therefore only be performed in appropriately selected patients with a high suspicion of fresh fractures. As radiography is the first-choice imaging examination for the diagnosis of OLVF, improving screening accuracy with radiographs will optimize the decision of whether an MRI is necessary. This study aimed to develop a method to automatically classify lumbar vertebrae (LV) conditions such as normal, old, or fresh OLVF using deep learning methods with radiography. A total of 3481 LV images for training, validation, and testing and 662 LV images for external validation were collected. Visual evaluation by two radiologists determined the ground truth of LV diagnoses. Three convolutional neural networks were ensembled. The accuracy, sensitivity, and specificity were 0.89, 0.83, and 0.92 in the test and 0.84, 0.76, and 0.89 in the external validation, respectively. The results suggest that the proposed method can contribute to the accurate automatic classification of LV conditions on radiography.

## 1. Introduction

Osteoporotic lumbar vertebral fracture (OLVF) is one of the most common complications in osteoporotic patients. The main factors associated with OLVF are a decrease in bone density, bone quality deterioration, and bone microstructure degeneration caused by osteoporosis [1,2]. Since the risk of osteoporosis increases with age, the number of patients with osteoporosis and those who develop OLVF will continue to increase as life expectancy increases [3,4,5].

As OLVF progresses, chronic severe pain, decreased vertebral body height, a round back, gait difficulty, decreased pulmonary function, and increased mortality significantly lead to a decrease in activities of daily living (ADL) and quality of life (QOL) [6,7]. Although the progression of these symptoms is often stabilized in patients with old OLVF, the prognosis of fresh OLVF is poor unless appropriate intervention is provided early. Therefore, when OLVF is confirmed, it is crucial to confirm the diagnosis of fresh OLVF as early as possible, relieve pain, and prevent the progression of crushing to maintain ADL and QOL in fresh OLVF patients [8,9,10].

Generally, magnetic resonance imaging (MRI) is used to determine whether OLVF is old or fresh [11]. The presence of fresh OLVF is indicated by low signal intensity on T1-weighted images and high signal intensity on T2-weighted and short tau inversion recovery (STIR) images, which reflect vertebral edema. MRI is a highly sensitive and specific method to determine whether an OLVF is old or fresh [12]. MRI can also detect subclinical fractures and identify the site of injury. MRI is an important tool for diagnosing OLVF because it allows for more detailed treatment strategy decisions [13,14]. On the other hand, MRI is a high-cost exam that burdens patients with severe back pain by forcing them to maintain their body position during long examinations. MRI also has drawbacks, such as the difficulty of emergency examinations and the limited number of examinations that can be performed in a day [11,13,15,16]. MRI should therefore only be performed on appropriately selected patients with a high suspicion of fresh OLVF who are most likely to require therapeutic intervention [15].

Currently, when fresh OLVF is suspected, the first-choice imaging examination is radiography, which is superior in image acquisition time, simplicity, low exposure dose, and low cost [10,17,18]. Radiography can screen for the presence of OLVF by capturing morphological changes in the vertebral body. However, due to its characteristics, it is difficult to diagnose fine fractures with few morphological changes immediately after onset and to accurately determine the stage of OLVF even when a decrease in vertebral height is observed [13,15,19,20]. It is also known that the probability of developing multiple OLVFs is higher in patients who have developed OLVF once in the past [21,22]. In patients with multiple OLVFs, especially when there are both old and fresh fractures in a radiograph, it is more difficult to visually identify the causative vertebrae from their morphology and determine whether MRI is indicated. In such cases, an old or fresh OLVF diagnosis may significantly depend on the physician’s experience and ability. To optimize the decision of the indication for MRI, it is therefore necessary to improve the accuracy of OLVF screening in radiography and develop a new automatic evaluation method that is not easily influenced by differences in the physicians’ experience and ability.

In recent years, deep learning (DL) methods based on network structures called convolutional neural networks (CNNs) have been used to solve various problems in the field of medical imaging. One of the most important features is high image classification performance based on high feature extraction capability [23,24]. In this study, we utilize DL methods of object detection and 3-class classification based on CNNs. The object detection algorithm used in this study is ‘you only look once’ at version 5 (YOLOv5). It has been reported that YOLO has high efficiency and detection accuracy and meets the requirements for use in clinical practice [25,26,27,28].

In a previous study that attempted to identify fresh vertebral compression fractures using radiography, the CNN model was designed for a 2-class classification of fresh and old fractures but could not identify normal vertebrae [29]. Therefore, it was necessary to manually select vertebrae with suspected fractures before applying the CNN model. To the best of our knowledge, this is the first study to be able to classify not only fresh and old vertebrae but also normal vertebrae. A 3-class classification allows all vertebrae to be included without prior selection. This reduces the burden of selecting the target vertebrae and the risk of missing a fresh fracture in another vertebra other than the target vertebra.

We have developed an efficient evaluation method with high detection capability by combining radiography’s quick image acquisition with CNNs high image classification performance. The use of CNNs to accurately detect fresh OLVF in previously difficult cases to visually evaluate with radiography makes it possible to more accurately determine the indication for further examination using MRI, regardless of the experience or specialty of the attending physician. This study, therefore, aims to evaluate our method to automatically determine the presence of OLVF and classify old and fresh OLVF using a CNN model with radiographs.

## 2. Materials and Methods

**Figure 1:**
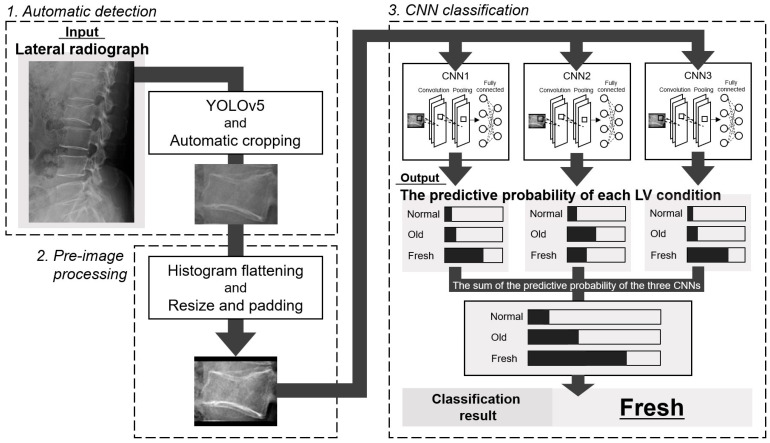
Overview of this study. LV means lumber vertebrae. Normal, normal vertebra; Old, old osteoporotic lumbar vertebral fractures; Fresh, fresh osteoporotic lumbar vertebral fractures.

The overview of this study is shown in Figure 1. In this study, we first automatically detected each lumbar vertebra from lateral radiographs. Then, after preliminary image processing, each vertebra was classified into normal, old, or fresh OLVF using three CNNs. Accuracy evaluation using external data was also performed for both (detection and classification) DL methods.

This manuscript was constructed according to the Standards for Reporting Diagnostic Accuracy Studies (STARD) 2015 guidelines [30].

### 2.1. Subjects

Patients who underwent both lumbar vertebrae radiography and MRI were included in this study. Radiographs for our DL method were collected at two institutions (Institutions 1 and 2). Institutional review boards approved this study in both institutions (institution 1: No. 20-00457, September 2020, and institution 2: No. 020-0342, March 2021). Informed consent in this retrospective study was obtained from all subjects by the opt-out method.

We collected lateral lumbar vertebral radiographs. In Institution 1, if anterior and posterior flexion imaging were acquired in addition to lateral lumbar vertebrae radiography, both images were also included. They were used as sample images to detect each lumbar vertebra automatically. Each lumbar vertebra image, after automatic cropping from lateral radiographs, was used as a sample image for CNN classification. Furthermore, in patients with thoracic vertebrae imaging, lumbar vertebrae included in the lateral thoracic vertebrae radiographs were also used for CNN classification.

In Institution 1, 523 consecutive patients with suspected OLVF who underwent radiography and MRI from March 2010 to December 2021 were included. In patients with fresh OLVF, lumbar vertebrae MRI was performed with a mean of 3.7 ± 17.0 days after radiography. Each vertebra (the first to the fifth lumbar vertebrae) was blinded to patient information and independently visually evaluated by two radiologists (14 and 12 years of experience) and classified as normal, old, or fresh OLVF. When the evaluation by two radiologists did not agree, the classification group was determined by consensus. In addition, for lumbar vertebrae that were determined to be fresh, the radiologists evaluated whether they were OLVF or pathological fractures. Pathological fractures are those resulting from bone weakness caused by primary or metastatic bone tumors.

Ninety-three patients whose visual evaluation showed that all the lumbar vertebrae from the first to the fifth were normal were excluded from this study because they may be different in the presence or absence of osteoporosis, that is, in the background of bone density, compared to patients with OLVF. To further improve the accuracy of the determination of freshness, the date of injury onset was confirmed for all patients with OLVF judged fresh in the visual evaluation. Of the 430 patients with fresh or old OLVF in one or more vertebrae, 12 fresh and three old OLVF patients were excluded due to exclusion reasons such as severe crush, foreign substance, poor positioning, poor image quality, injury onset date unknown, or only pathological fracture (if there was OLVF other than pathological fractures, those patients were included). In this study, the criterion for severe crush was a more than 40% reduction in post-fracture vertebral body height compared to pre-fracture vertebral body height, based on Genant’s criteria [31].

**Figure 2:**
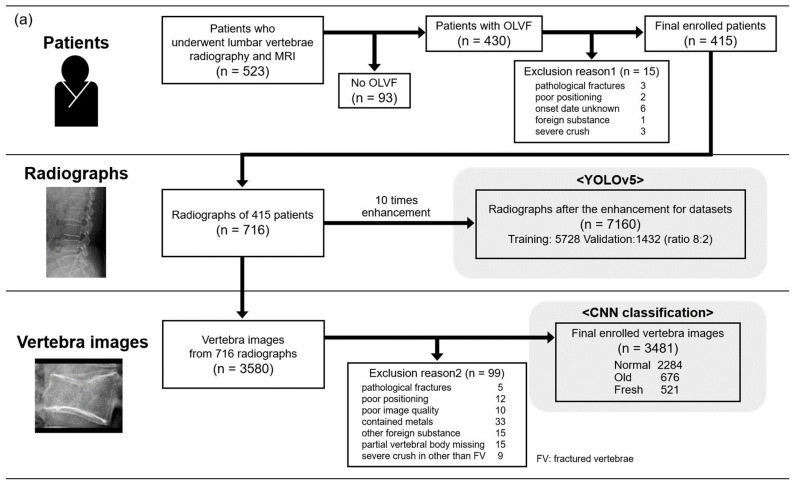

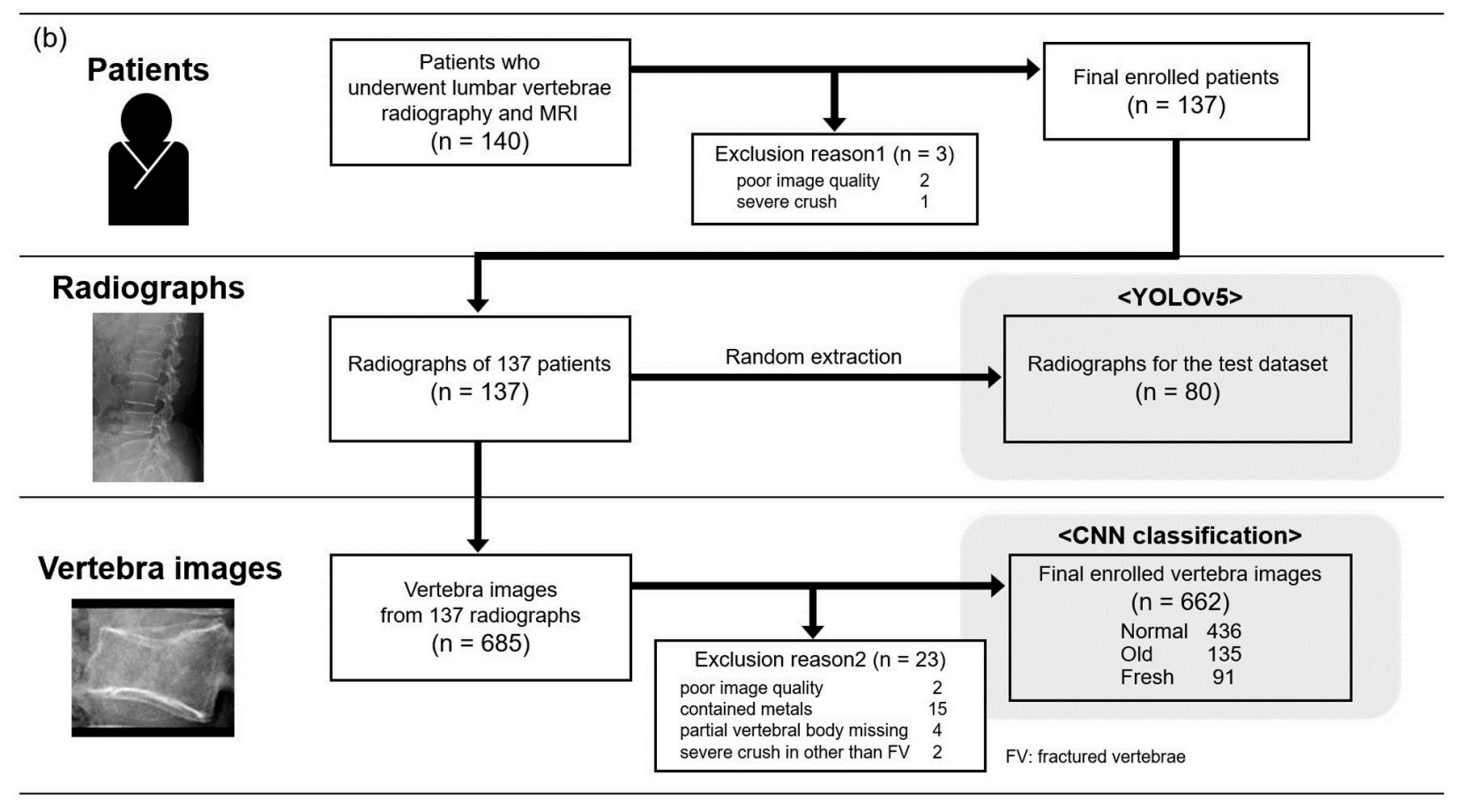
The number of subjects in Institutions 1 (**a**) and 2 (**b**). Normal, normal vertebra; Old, old osteoporotic lumbar vertebral fractures; Fresh, fresh osteoporotic lumbar vertebral fractures.

Exclusion reason 1 focused only on the condition of the fractured vertebrae in radiographs, while exclusion reason 2 covered all images of each vertebra after cropping from radiographs. As a result, 415 subjects were employed in this study (Figure 2a).

In Institution 2, 140 patients who underwent radiography and MRI from January 2011 to December 2021 and were diagnosed with OLVF in MRI interpretation reports by radiologists in daily practice were included in this study. In patients with fresh OLVF, lumbar vertebrae MRI was performed with a mean of 8.1 ± 10.9 days after radiography was acquired. After image collection, two fresh and one old OLVF patient were excluded based on the same exclusion criteria as in Institution 1. As a result, 137 subjects were employed in this study (Figure 2b).

### 2.2. Image Acquisition

In Institution 1, radiographs were acquired using either the flat panel detector (FPD) of the CALNEO Smart C77 or the CALNEO MT (FUJIFILM Medical Co., Ltd., Tokyo, Japan). CALNEO Smart C77 uses CsI scintillators, and CALNEO MT uses GOS scintillators. For both FPDs, a real grid (8:1 grid ratio) (Mitaya Manufacturing Co., Ltd., Saitama, Japan) was used for scattered radiation removal instead of a scattered radiation correction process such as a virtual grid (FUJIFILM Medical Co., Ltd., Tokyo, Japan). The acquired image size was 10 × 12 inches, the pixel size was 0.15 mm, and the grayscale depth was 14 bits. The source-to-image receptor distance (SID) was 110 cm, the tube voltage was 85 kV, and the current value was automatically determined by the auto exposure control (AEC) system according to the patient’s body thickness. The X-ray generator was RAD Speed Pro (SHIMADZU Corporation, Kyoto, Japan). All MRI images were acquired using a 1.5 Tesla MRI system from Ingenia (Philips Healthcare, Best, The Netherlands).

In Institution 2, radiographs were acquired using either of three FPDs. The X-ray generator and FPD in each room are shown in Table S1. The scattered radiation removal was performed on a real grid with a grid ratio of 8:1 or 10:1. The acquired image size was 14 × 17 inches, the pixel size was 0.15 mm, and the grayscale depth was 14 bits. The SID was 130 cm, the tube voltage was 90 kV, and the AEC system automatically determined the current value. An MRI was performed in a total of five rooms. The imaging equipment and magnetic field strength in each room are shown in Table S2.

### 2.3. Vertebral Body Detection with You Only Look Once

The automatic object detection algorithm used in this study was YOLOv5. There are five main models available to the public: YOLOv5 n/s/m/l/x. The main differences between versions are the automatic detection accuracy and calculation load. In this study, YOLOv5x (the largest) was selected and finetuned in training. Training and validation were conducted using 5728 training images and 1432 validation images (8:2 ratio), with a 10-fold augmentation of 716 radiographs from 415 patients in Institution 1. The image augmentation was performed using Imgaug, a Python library. Details of the image augmentation process are shown in Table 1. A total of six image processing steps were combined to create the processed image. The intensity of each process was randomly determined between the maximum and minimum values. Eighty radiographs were randomly selected out of 137 radiographs from 137 patients in Institution 2 and used for the test. One radiological technologist manually set the ground truth bounding box using the free software labelImg. All sample images were converted to 8-bit PNG images of 640 × 640 pixels. The following parameters were determined by hyperparameter evolution, a method of hyperparameter optimization using a genetic algorithm included in the YOLOv5 system: epochs, 300; batch size, 4; initial learning rate, 0.00967; momentum, 0.92755; weight decay, 0.00057.

### 2.4. Sample Creation

**Figure 3:**
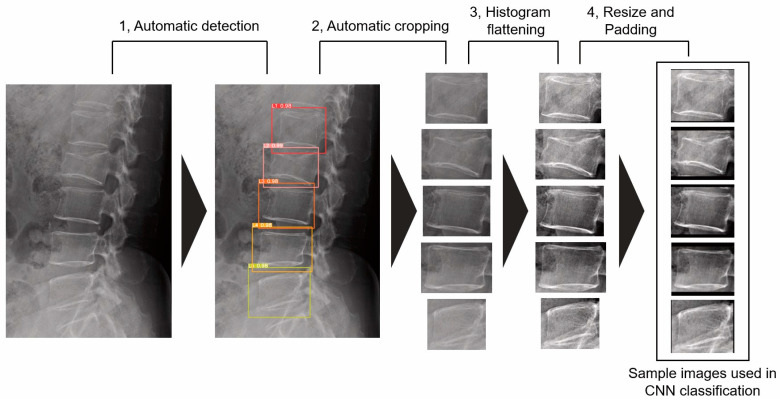
The flow of sample image creation The vertebral number and the confidence value of automatic detection accompany bounding boxes detected by YOLOv5.

Each lumbar vertebra was automatically cropped based on the bounding box coordinates detected by YOLOv5. After cropping, a histogram flattening process was applied. The image resolution was resized and padded as necessary to 166 (W) × 140 (H) pixels (Figure 3). In this sample creation phase, 99 and 23 sample images were excluded from Institutions 1 and 2, respectively, due to the adverse conditions shown in Figure 2.

### 2.5. Datasets Creation and CNN Classification

In this study, a 3-class CNN classification was performed. All sample images are classified into normal, old, or fresh OLVF groups.

**Figure 4:**
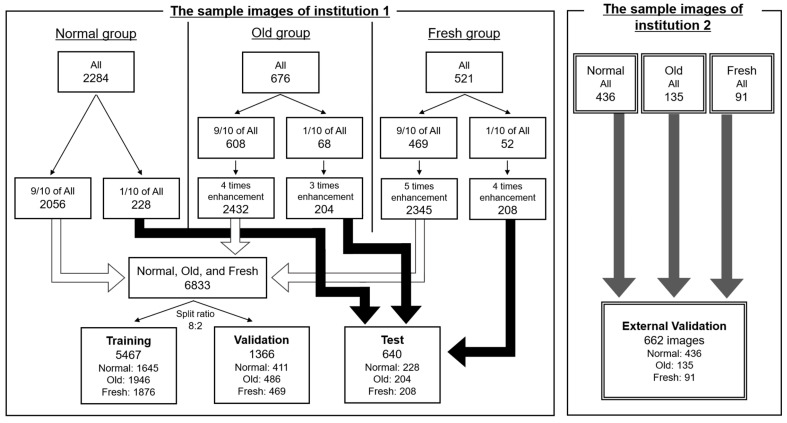
Datasets creation for the CNN classification. Normal, normal vertebra; Old, old osteoporotic lumbar vertebral fractures; Fresh, fresh osteoporotic lumbar vertebral fractures.

In Institution 1, 228, 68, and 52 sample images, or about 1/10 of the total images in the normal, old, and fresh OLVF groups, were divided, and the number of divided old and fresh OLVF images was tripled and quadrupled to resolve the imbalance in the number of images among each group. As a result, 228, 204, and 208 sample images were prepared in the normal, old, and fresh OLVF groups, respectively, as the test dataset images. After the test dataset image division, a total of 6833 sample images (2056 normal, 2432 old, and 2345 fresh sample images) were divided in the ratio of training 8: validation 2. In Institution 2, no augmentation of the number of sample images was performed. As a result, 436, 135, and 91 sample images were prepared in the normal, old, and fresh OLVF groups, respectively. The sample images in Institution 2 were used for external validation (Figure 4).

**Figure 5:**
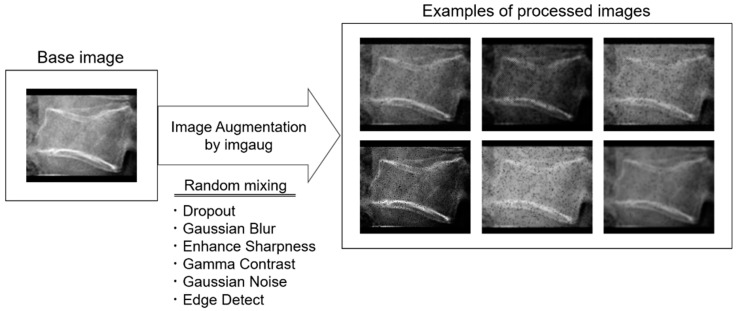
Examples of processed images by imgaug. A total of six image processing steps were combined to create the processed image.

A total of four datasets were prepared: training, validation, test, and external validation. The training datasets were used to create the model to automatically determine the presence of OLVF and classify old and fresh OLVF, while the validation datasets were used to adjust the hyperparameters. The test dataset was used to evaluate the classification performance by using images with the same characteristics as those used for training and validation, while external validation was an evaluation of the classification performance on completely unknown images. The important point is that the images in the test and external validation datasets were not used for training or validation. The robustness of the model created in this study was evaluated in more detail by also performing external validation. The parameter settings were the same as in YOLOv5 training (Table 1). Examples of processed images are shown in Figure 5.

The CNNs output the probability that the input image is normal, old, or fresh OLVF. The class with the highest sum of the predictive probabilities output by three CNNs for each classification group was determined as the result of the CNN classification.

### 2.6. CNN Model

An ensemble model using three CNNs was employed in this study. The CNNs used were Resnet-50, DenseNet-161, and Res-NeXt-50. Each CNN was pre-trained with initial weights trained on ImageNet, a large image dataset on Neural Network Console (NNC) (Sony Network Communications Inc., Tokyo, Japan). Each pre-trained CNN model is available at https://nnabla.readthedocs.io/en/latest/python/api/models/imagenet.html (accessed on 15 May 2021). Before the training process, three layers were inserted just below the input layer in each CNN. The first is the “Broadcast” layer to change the color channel of input images from 1 to 3. This allows the use of grayscale images for input in CNN trained with color images. The second is the “MulScalarX” layer to divide pixel values by 255 to normalize pixel values. The third is the “ImageAugmentation” layer to pseudo-enhance the number of sample images to reduce overfitting due to the insufficient number of images. This image augmentation process included scaling, rotation, brightness, and contrast changes. The details of each enhancement process are shown in Table S3. The learning rates of Resnet-50, DenseNet-161, and ResNeXt-50 were set to 0.01, 0.001, and 0.01, respectively. The following training parameters were common to all three CNNs: 100 epochs; batch size, 4; optimizer, Nesterov.

### 2.7. Deep Learning

The development environment for DL in this study was Windows 10 Pro (64bit), Intel^®^ Core™ i7-10700KF, NVIDIA^®^ GeForce RTX™ 3080, Python version 3.8.8, PyTorch version 1.8.1, and NNC version 2.1.0.

### 2.8. Statistical Analysis

Quantitative data were expressed as the mean and standard deviation. Interobserver and intraobserver visual assessment for the vertebrae condition classification were evaluated by weighted kappa values using the Landis and Koch criteria (0.0–0.2: slight agreement, 0.21–0.40: fair agreement, 0.41–0.60: moderate agreement, 0.61–0.80: substantial agreement, 0.81–1.0: almost perfect agreement) [32]. The second visual evaluation for intraobserver calculation was performed more than one month after the first assessment, and radiographs of 50 randomly selected patients from a total of 523 patients were used. All weighted kappa values were calculated using R (version 4.2.0) and the package “irr” (ver0.84.1).

The detection accuracy of the YOLOv5 model developed in this study was evaluated by the mean average precision (mAP). The mAP is the score representing the degree of agreement between the coordinates of the detected bounding box and the ground truth bounding box and is defined as follows:

$$mAP=\frac{1}{n}\sum_{k=1}^{k=n}{AP}_{k}$$

APk is the average precision of class k and n represents the number of classes. In this study, the mAP (0.5) and the mAP (0.5: 0.95) were used as evaluation indices. The mAP (0.5) is the mAP when the intersection over union (IoU) is set to 0.5, and the mAP (0.5: 0.95) is the average of the mAP obtained by changing IoU from 0.5 to 0.95 in 0.05 steps. IoU is an index indicating the overlap degree between the detected bounding box by YOLO and the ground truth bounding box and is calculated by dividing the common part of the two regions by the sum set.

For CNN classification, we evaluated the classification performance using 5-fold cross-validation. In this method, the datasets are divided into five groups, one of which is the validation data, and the remaining four groups are the training data, and the classification performance is evaluated. All five groups are assigned to the validation data one at a time. Training and validation were performed five times in each CNN per cross-validation component, for a total of 15 results.

The accuracy, sensitivity, specificity, false positive rate, and false negative rate were calculated for the classification performance. These classification performances were calculated by considering either normal, old, or fresh OLVF as positive and the others as negative. The name of the group considered positive was appended to each classification performance. The overall classification performance is the average of the values calculated when each group is considered positive. For example, when normal is considered positive, old and fresh OLVF are considered negative, and the accuracy is shown as accuracy_normal._ Accuracy_all_ is the average of accuracy_normal_, accuracy_old_, and accuracy_fresh_.

In addition, receiver operating characteristic (ROC) curves were plotted, and the area under the curve (AUC) values were calculated. They were plotted and calculated by *scikit-learn*, one of the Python libraries.

A total of 95% confidence intervals (CI) for the classification performance and AUC were calculated using scikit-learn and statsmodels in the Python libraries, respectively.

## 3. Results

In Institution 1, a total of 716 lateral lumber vertebrae radiographs of 415 OLVF patients were employed. The subjects consisted of 280 fresh OLVF patients with a mean age of 78.5 ± 11.4 years and 135 old OLVF patients with a mean age of 77.1 ± 9.3 years. No significant difference in mean age between fresh and old OLVF patients was observed (*p* > 0.05). In 280 fresh OLVF patients, radiography in 200 patients (71.4%) was performed within 14 days from the injury onset.

In Institution 2, a total of 137 lateral lumber vertebrae radiographs of 137 OLVF patients were employed. The subjects consisted of 77 fresh OLVF patients with a mean age of 69.6 ± 13.7 years and 60 old OLVF patients with a mean age of 70.8 ± 11.1 years. No significant difference in mean age between fresh and old OLVF patients was observed (*p* > 0.05). In 77 fresh OLVF patients, radiography was performed in 48 patients (62.3%) within 14 days from the injury onset.

### 3.1. Agreement Rate in the Visual Evaluation of Each Vertebral Body

The interobserver agreement value for visual evaluation by two radiologists was 0.801. The intraobserver agreement values for raters 1 and 2 were 0.821 and 0.861, respectively. The agreement in all evaluations was almost perfect. Consensus was required in 141 of 523 cases because of inconsistent evaluation of at least one vertebra.

### 3.2. YOLOv5

The detection performance of YOLOv5 was mAP (0.5) of 0.995 and mAP (0.5: 0.95) of 0.993 for the validation dataset and mAP (0.5) of 0.982 and mAP (0.5: 0.95) of 0.835 for the test dataset in terms of detection for lumber vertebrae. Using automatic cropping based on the bounding box detected by YOLOv5, vertebra images of 2284 normal, 676 old, and 521 fresh OLVF were produced in Institution 1. Similarly, vertebra images of 436 normal, 135 old, and 91 fresh OLVF were produced in Institution 2. The breakdown of vertebral body numbers is shown in Table 2.

### 3.3. Classification Performance by CNNs

**Figure 6:**
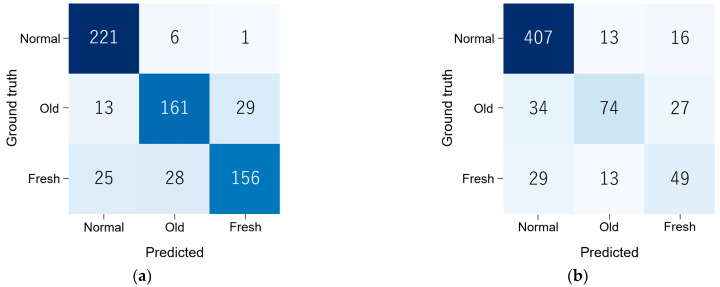
The confusion matrix in CNN classification in the test (**a**) and external validation (**b**) datasets. Normal, normal vertebra; Old, old osteoporotic lumbar vertebral fractures; Fresh, fresh osteoporotic lumbar vertebral fractures.

**Figure 7:**
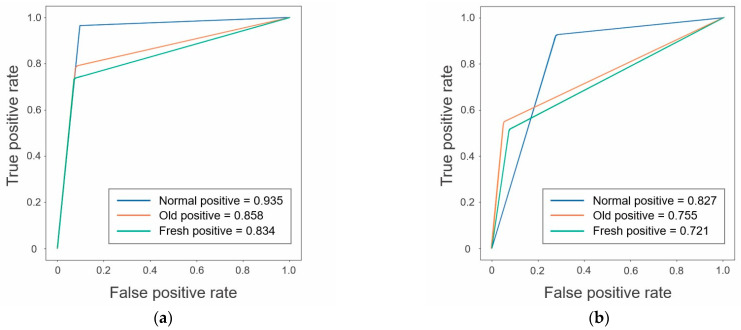
ROC curve for the test (**a**) and external validation (**b**) datasets. Normal, normal vertebra; Old, old osteoporotic lumbar vertebral fractures; Fresh, fresh osteoporotic lumbar vertebral fractures.

The confusion matrix in this classification is shown in Figure 6. The classification performance was as follows: The accuracy_all_, sensitivity_all_, specificity_all_, false positive rate_all_, false negative rate_all_, and AUC_all_ was 0.894 [CI: 0.870–0.917], 0.836 [CI: 0.790–0.882], 0.920 [CI: 0.894–0.946], 0.161 [CI: 0.111–0.211], 0.077 [CI: 0.053–0.100], and 0.876 [CI: 0.848–0.903] in the test dataset, and 0.867 [CI: 0.841–0.892], 0.674 [CI: 0.603–0.743], 0.866 [CI: 0.832–0.898], 0.288 [CI: 0.217–0.363], 0.111 [CI: 0.079–0.144], and 0.768 [CI: 0.726–0.811] in the external validation dataset, respectively. A high accuracy_all_ of 0.86 or higher in both datasets were achieved. The ROC curves for each dataset are shown in Figure 7.

The classification performances in both datasets were summarized in Table 3.

## 4. Discussion

In this retrospective study, we attempted to develop an automatic method to detect OLVF and classify old and fresh OLVF by creating a CNN model with lateral lumbar vertebrae radiographs. This is the first study to validate the generalization performance of 3-class OLVF classification using images from multiple facilities. Some images in the external validation dataset differed from those used in the training and validation regarding imaging conditions that affect image quality, such as the tube voltage and grid ratio. The fact that our method is effective even for such images may suggest that this model is quite versatile.

Objectivity must be ensured in the sample creation process. In this study, YOLOv5 was applied to the sample creation process. This eliminates human bias caused by manual procedures and reduces sample creation time. To the best of our knowledge, though one study attempted to identify fresh vertebral fractures on radiography using a CNN, that study had the limitation that the ROIs to extract the target vertebrae were manually drawn [29], which has been overcome in our study.

A study reported by Strickland et al. (2023) [33] to determine fresh OLVF based on findings on radiography showed a sensitivity of 52% and a specificity of 95%. Langdon et al. stated that it is not possible to distinguish between fresh and old fractures on radiographs [16]. As described above, the human evaluation to determine fresh OLVF using radiographs is very difficult. The sensitivity of 84% and 67% and specificity of 92% and 87% (test/external validation) were achieved in the proposed method. Compared to the diagnostic accuracy by radiographs alone as reported by Strickland et al., the sensitivity was at least 15% higher and the specificity was comparable even in the more difficult situation, the classification in the external validation dataset. We believe that the proposed method combining CNN with radiography has high classification and generalization performance and would further improve the usefulness of radiography.

The implementation of this method may benefit both the physician and the patient. In clinical practice, physicians carefully observe each vertebral body on radiographs to determine the diagnosis. By referring to the objective and consistent classification results provided by the CNN model developed in this study, physicians can reduce the burden associated with the evaluation of radiography and make a diagnosis and therapeutic strategy in a shorter time. In addition, the proposed method’s improved fresh OLVF screening accuracy prevents missed fresh OLVF and reduces unnecessary MRI imaging. As a result, it will enable a more efficient selection of patients who require close examination by MRI. Furthermore, the proposed method requires only radiography, one of the most widely used imaging exams. This greatly benefits patients by allowing them to receive high-accuracy screening at any given facility. The proposed method may be more effective when accurate diagnosis is difficult due to the co-existence of old and fresh OLVFs on radiographs, even if the physicians are inexperienced in evaluating OLVFs.

The current challenge for clinical practice is that the target lumbar vertebrae images must be extracted from the image storage server and transferred to a device for machine learning each time. The other issue is that showing physicians the basis for the CNN classification is impossible.

The limitations of this study are as follows: First, cases of lumber vertebrae with deformation/crush, strong scoliosis, and metal material implantation were excluded. If the lumber vertebrae with such characteristics are input into the CNN created in this study, it may be unable to output the correct diagnosis because it was not trained on such images. Second, since this study targeted OLVF, it is unclear whether high classification accuracy can be guaranteed for pathological fractures caused by bony metastasis. It may be difficult to utilize this CNN at facilities with many pathological fracture cases. Third, the number of sample images is limited. To create a more accurate and versatile network, it is necessary to increase the number of samples, collect image data from more facilities, and conduct training using images with various characteristics.

## 5. Conclusions

The proposed CNN-based method demonstrated high performance in determining the presence of OLVF and classifying old or fresh OLVF on radiography. Utilizing objective classification results from our CNN is expected to improve the accuracy of fresh OLVF screening. This may lead to appropriate decisions on the indication for close examination with MRI.

## Figures and Tables

**Table 1:** Details of the image augmentation process by imgaug.

	Parameter Range	
	Min	Max
Dropout	0.0	0.1
Gaussian Blur	0.0	0.5
Enhance Sharpness	0.0	2.5
Gamma Contrast	0.5	2.0
Gaussian Noise	0	0.05 × 255
Edge Detect	0.05	0.3

**Table 2:** The breakdown of each vertebral body number in this study.

	Institution 1			Institution 2		
	Normal	Fresh	Old	Normal	Fresh	Old
The number of all vertebra images	2284	521	676	436	91	135
L1 (%)	322 (14.1)	175 (33.6)	211 (31.2)	63 (14.4)	26 (28.6)	43 (31.9)
L2 (%)	467 (20.4)	118 (22.6)	124 (18.3)	83 (19.0)	23 (25.3)	27 (20.0)
L3 (%)	464 (20.3)	106 (20.3)	131 (19.4)	87 (20.0)	19 (20.9)	28 (20.7)
L4 (%)	486 (21.3)	76 (14.6)	129 (19.1)	97 (22.2)	15 (17.0)	23 (17.0)
L5 (%)	545 (23.9)	46 (8.8)	81 (12.0)	106 (24.3)	8 (8.8)	14 (10.4)

Normal, normal vertebra; Old, old osteoporotic lumbar vertebral fractures; Fresh, fresh osteoporotic lumbar vertebral fractures.

**Table 3:** The summary of each classification performance.

	Test				External Validation			
		The Group Considered Positive				The Group Considered Positive		
	All	Normal	Old	Fresh	All	Normal	Old	Fresh
Accuracy	0.894	0.930	0.880	0.871	0.867	0.861	0.868	0.872
Sensitivity	0.836	0.968	0.791	0.748	0.674	0.933	0.550	0.538
Specificity	0.920	0.908	0.922	0.930	0.866	0.722	0.950	0.925
False positive rate	0.161	0.146	0.175	0.163	0.288	0.134	0.264	0.465
False negative rate	0.077	0.019	0.096	0.115	0.111	0.152	0.108	0.074
AUC	0.876	0.935	0.858	0.834	0.768	0.827	0.755	0.721

Normal, normal vertebra; Old, old osteoporotic lumbar vertebral fractures; Fresh, fresh osteoporotic lumbar vertebral fractures.

## Data Availability

All relevant data are not publicly available for privacy reasons.

## Supplementary Materials

The following supporting information can be downloaded at: https://www.mdpi.com/article/10.3390/jimaging9090187/s1. Table S1. Radiography imaging equipment used in institution 2; Table S2. MRI imaging equipment and magnetic field strength used in institution 2; Table S3. Details of the image enhancement process by the ImageAugmentation layer on NNC.

## References

[1] Xu W., Wang S., Chen C., Li Y., Ji Y., Zhu X., Li Z. (2018). Correlation analysis between the magnetic resonance imaging characteristics of osteoporotic vertebral compression fractures and the efficacy of percutaneous vertebroplasty: A prospective cohort study. BMC Musculoskelet. Disord..

[2] Su W.C., Wu W.T., Peng C.H., Yu T.C., Lee R.P., Wang J.H., Yeh K.T. (2022). The Short-Term Changes of the Sagittal Spinal Alignments After Acute Vertebral Compression Fracture Receiving Vertebroplasty and Their Relationship with the Change of Bathel Index in the Elderly. Geriatr. Orthop. Surg. Rehabil..

[3] Manhard M.K., Nyman J.S., Does M.D. (2017). Advances in imaging approaches to fracture risk evaluation. Transl. Res..

[4] Tan E., Wang T., Pelletier M.H., Walsh W.R. (2016). Effects of cement augmentation on the mechanical stability of multilevel spine after vertebral compression fracture. J. Spine Surg..

[5] Zhang H., Xu C., Zhang T., Gao Z., Zhang T. (2017). Does Percutaneous Vertebroplasty or Balloon Kyphoplasty for Osteoporotic Vertebral Compression Fractures Increase the Incidence of New Vertebral Fractures? A Meta-Analysis. Pain Physician.

[6] Jin C., Xu G., Weng D., Xie M., Qian Y. (2018). Impact of Magnetic Resonance Imaging on Treatment-Related Decision Making for Osteoporotic Vertebral Compression Fracture: A Prospective Randomized Trial. Med. Sci. Monit..

[7] Cheng J., Muheremu A., Zeng X., Liu L., Liu Y., Chen Y. (2019). Percutaneous vertebroplasty vs balloon kyphoplasty in the treatment of newly onset osteoporotic vertebral compression fractures: A retrospective cohort study. Medicine.

[8] Suzuki N., Ogikubo O., Hansson T. (2009). The prognosis for pain, disability, activities of daily living and quality of life after an acute osteoporotic vertebral body fracture: Its relation to fracture level, type of fracture and grade of fracture deformation. Eur. Spine J..

[9] Shigenobu K., Hashimoto T., Kanayama M., Ohha H., Yamane S. (2019). The efficacy of osteoporotic treatment in patients with new spinal vertebral compression fracture pain, ADL, QOL, bone metabolism and fracture-healing—In comparison with weekly teriparatide with bisphosphonate. Bone Rep..

[10] Li Y.-C., Chen H.-H., Lu H.H.-S., Wu H.-T.H., Chang M.-C., Chou P.-H. (2021). Can a Deep-learning Model for the Automated Detection of Vertebral Fractures Approach the Performance Level of Human Subspecialists?. Clin. Orthop. Relat. Res..

[11] Lenski M., Büser N., Scherer M. (2017). Concomitant and previous osteoporotic vertebral fractures. Acta Orthop..

[12] Choi W.H., Oh S.H., Lee C.J., Rhim J.K., Chung B.S., Hong H.J. (2012). Usefulness of SPAIR Image, Fracture Line and the Adjacent Discs Change on Magnetic Resonance Image in the Acute Osteoporotic Compression Fracture. Korean J. Spine.

[13] Marongiu G., Congia S., Verona M., Lombardo M., Podda D., Capone A. (2018). The impact of magnetic resonance imaging in the diagnostic and classification process of osteoporotic vertebral fractures. Injury.

[14] Lin H.H., Chou P.H., Wang S.T., Yu J.K., Chang M.C., Liu C.L. (2015). Determination of the painful level in osteoporotic vertebral fractures—Retrospective comparison between plain film, bone scan, and magnetic resonance imaging. J. Chin. Med. Assoc..

[15] Langdon J., Way A., Heaton S., Bernard J., Molloy S. (2010). Vertebral compression fractures—New clinical signs to aid diagnosis. Ann. R. Coll. Surg. Engl..

[16] Bierry G., Venkatasamy A., Kremer S., Dosch J.C., Dietemann J.L. (2014). Dual-energy CT in vertebral compression fractures: Performance of visual and quantitative analysis for bone marrow edema demonstration with comparison to MRI. Skeletal Radiol..

[17] Kim D.H., Jeong J.G., Kim Y.J., Kim K.G., Jeon J.Y. (2021). Automated Vertebral Segmentation and Measurement of Vertebral Compression Ratio Based on Deep Learning in X-Ray Images. J. Digit. Imaging.

[18] Kim K.C., Cho H.C., Jang T.J., Choi J.M., Seo J.K. (2021). Automatic detection and segmentation of lumbar vertebrae from X-ray images for compression fracture evaluation. Comput. Methods Programs Biomed..

[19] Diekhoff T., Engelhard N., Fuchs M., Pumberger M., Putzier M., Mews J., Makowski M., Hamm B., Hermann K.A. (2019). Single-source dual-energy computed tomography for the assessment of bone marrow oedema in vertebral compression fractures: A prospective diagnostic accuracy study. Eur. Radiol..

[20] Maselli F., Rossettini G., Viceconti A., Testa M. (2019). Importance of screening in physical therapy: Vertebral fracture of thoracolumbar junction in a recreational runner. BMJ Case Rep..

[21] Suzuki N., Ogikubo O., Hansson T. (2010). Previous vertebral compression fractures add to the deterioration of the disability and quality of life after an acute compression fracture. Eur. Spine J..

[22] Oudshoorn C., Hartholt K.A., Zillikens M.C., Panneman M.J., van der Velde N., Colin E.M., Patka P., van der Cammen T.J. (2012). Emergency department visits due to vertebral fractures in the Netherlands, 1986–2008: Steep increase in the oldest old, strong association with falls. Injury.

[23] Cao Y., Yu J., Zhang H., Xiong J., Luo Z. (2022). Classification of hepatic cavernous hemangioma or hepatocellular carcinoma using a convolutional neural network model. J. Gastrointest. Oncol..

[24] Halme H.L., Ihalainen T., Suomalainen O., Loimaala A., Mätzke S., Uusitalo V., Sipilä O., Hippeläinen E. (2022). Convolutional neural networks for detection of transthyretin amyloidosis in 2D scintigraphy images. EJNMMI Res..

[25] Guo G., Zhang Z. (2022). Road damage detection algorithm for improved YOLOv5. Sci. Rep..

[26] Chen S., Duan J., Wang H., Wang R., Li J., Qi M., Duan Y., Qi S. (2022). Automatic detection of stroke lesion from diffusion-weighted imaging via the improved YOLOv5. Comput. Biol. Med..

[27] Wang C., Zhang Y., Zhou Y., Sun S., Zhang H., Wang Y. (2023). Automatic detection of indoor occupancy based on improved YOLOv5 model. Neural Comput. Appl..

[28] Shi Y., Li J., Yu Z., Li Y., Hu Y., Wu L. (2022). Multi-Barley Seed Detection Using iPhone Images and YOLOv5 Model. Foods.

[29] Chen W., Liu X., Li K., Luo Y., Bai S., Wu J., Chen W., Dong M., Guo D. (2022). A deep-learning model for identifying fresh vertebral compression fractures on digital radiography. Eur. Radiol..

[30] Bossuyt P.M., Reitsma J.B., Bruns D.E., Gatsonis C.A., Glasziou P.P., Irwig L., Lijmer J.G., Moher D., Rennie D., de Vet H.C.W. (2015). STARD 2015: An updated list of essential items for reporting diagnostic accuracy studies. BMJ.

[31] Genant H.K., Wu C.Y., van Kuijk C., Nevitt M.C. (1993). Vertebral fracture assessment using a semiquantitative technique. J. Bone Miner. Res..

[32] Landis J.R., Koch G.G. (1977). The measurement of observer agreement for categorical data. Biometrics.

[33] Strickland C.D., DeWitt P.E., Jesse M.K., Durst M.J., Korf J.A. (2023). Radiographic assessment of acute vs chronic vertebral compression fractures. Emerg. Radiol..

