# Supplementary material for: A Deep Learning-Based Model for Classifying Osteoporotic Lumbar Vertebral Fractures on Radiographs: A Retrospective Model Development and Validation Study
Source: J Imaging. 2023 Sep 18;9(9):187. doi: 10.3390/jimaging9090187 (PMC10532676; doi:10.3390/jimaging9090187)
Supplement: Supplementary file 1 [file jimaging-09-00187-s001.zip › jimaging-2600412-supplementary.pdf]

Supplemental materials

Table S1. Radiography Imaging Equipment used in Institution 2

	Examination room1	Examination room2	Examination room3
**X-ray generator**	BENEO	RAD speed Pro	Radnext 80
	FUJIFILM Medical Corporation	SHIMADZU Corporation	FUJIFILM Healthcare Corporation
**X-ray detector (scintillator)**	BENEO(a-Se)	CALNEO MT(GOS)	CALNEO C 1717 wireless SQ (CsI)
	FUJIFILM Medical Corporation	FUJIFILM Medical Corporation	FUJIFILM Medical Corporation
**Scattered Radiation Correction**	Real Grid	Real Grid	Real Grid
Grid ratio	10:1	8:1	8:1
	Mitaya Corporation	Mitaya Corporation	Mitaya Corporation

Table S2. MRI imaging Equipment and Magnetic Field Strength used in Institution 2

	Examination room1	Examination room2	Examination room3	Examination room4	Examination room5
**MRI system**	MAGNETOM Avanto	Discovery MR750w 3.0T	TRILLIUM OVAL	Achieva dStream	Ingenia Elition 3.0T
	SIEMENS Healthcare	GE Healthcare	FUJIFILM Healthcare	Philips Healthcare	Philips Healthcare
**Magnetic field strength (T)**	1.5	3.0	3.0	1.5	3.0

Table S3. Details of the Image Enhancement Process by the ImageAugmentation Layer on NNC.

	Parameter Range	
	Min	Max
Image scaling	0.8	1.05
Image rotation	-20°	+20°
Brightness change	-0.2	+0.2
Contrast change	1/1.5	1.5
